# Relationship between Serum Bilirubin and Left Ventricular Hypertrophy in Patients with Essential Hypertension

**DOI:** 10.1371/journal.pone.0125275

**Published:** 2015-04-27

**Authors:** Tao Zhou, Xiaofang Chen, Zhanzhan Li, Lezhi Li

**Affiliations:** 1 Department of Cardiology, the Third Affiliated Hospital, Southern Medical University, Guangzhou, Guangdong Province, China; 2 Xiangya Nursing School, Central South University, Changsha, Hunan Province, China and Nursing Department, The Third Affiliated Hostipal Of Southern Medical Universtiy, Guangzhou, Guangdong Province, China; 3 Department of Epidemiology and Health Statistics, School of Public Health, Central South University, Changsha, Hunan Province, China; 4 Department of Nursing, the Second Affiliated Hospital, Xiangya Medical School, Central South University, Changsha, Hunan Province, China; Osaka University Graduate School of Medicine, JAPAN

## Abstract

**Background:**

Prospective studies have found low bilirubin levels were an important predictive factor of cardiovascular events. However, few have yet investigated possible association between serum bilirubin level and LVH in essential hypertension. The aim of the present study was to evaluate the relationship between serum bilirubin levels with LVH in newly diagnosed hypertension patients.

**Methods:**

The present study evaluated the relationship between serum total bilirubin level and left ventricle hypertrophy (LVH) in newly diagnosed hypertensive patients with a sample size of 344. We divided subjects into LVH group (n=138) and non-LVH group (n=206). Physical examination, laboratory tests and echocardiography were conducted. The multivariate logistic regression model was used to verify the independent association between RDW and LVH.

**Results:**

Our results found that patients with LVH had lower bilirubin levels than non-LVH ones. Stepwise multiple linear regression analysis showed total bilirubin level (B=-0.017, *P*=0.008) was negatively associated with left ventricle mass index (LVMI) even adjusting for some confounders. The multiples logistic regression found total bilirubin level was independently related with of LVH, as a protective factors (OR=0.91, *P*=0.010).

**Conclusion:**

As a routine and quick laboratory examination index, serum bilirubin may be treated as novel marker for evaluating LVH risk in hypertensive patients. Cohort study with larger sample size are needed.

## Introduction

Hypertension is an important public health issue worldwide [1] Hypertension could damage various target organs [2] and thus raise the risks of coronary heart disease, heart failure, chronic kidney disease (CKD) and stroke [3–5]. Left ventricle hypertrophy (LVH) is a common subclinical organ damage induced by hypertension. The prevalence of LVH among hypertensive patients is about 20%–40% [6]. LVH has been suggested as a validated marker indicating the mortality of cardiovascular diseases (CVDs) [7]. Therefore, identifying specific risk factors of LVH in hypertensive patients is quite important for reducing the incidence of cardiovascular events. Many common risk factors of LVH have been confirmed through epidemiologic research, such as obesity, old age, high blood pressure, and smoking status [8].

Now, growing attention has been paid to some serological indices, such as serum bilirubin level. As the final product of heme catabolism, bilirubin is anti-inflammatory and antioxidant in vitro and in vivo [9]. Epidemiologic evidence has shown that the increase in serum bilirubin level, even within normal range [10], is a protective factor of CVDs. A clinical study suggests that people with lower serum bilirubin levels are more likely to suffer from hypertension, diabetes and obesity [11]. Moreover, prospective studies also show that low bilirubin level is a main predictive factor of cardiovascular events, such as stroke, heart failure and coronary artery disease [12–14]. However, the possible association between serum bilirubin level and the occurrence of LVH in essential hypertensive patients has been rarely investigated. Bilirubin can suppress the oxidation of blood lipids including low-density lipoprotein (LDL), and the application of bilirubin can improve the marker of anti-oxidative stress [15, 16]. Therefore, we assume that high serum bilirubin level may be a protective factor of LVH in hypertensive patients. On this basis, serum bilirubin level examination can be conducted as a cheap routine test and as a potential predictor of LVH in newly-diagnosed hypertensive patients. The aim of the present study is to evaluate the relation between serum bilirubin level and the occurrence of LVH in newly-diagnosed hypertensive patients.

## Methods

### Study population

This cross-sectional study preliminarily involved 408 consecutive hypertensive patients who had not received any treatment before and were enrolled in the outpatient clinic of the Third Affiliated Hospital at Southern Medical University between October 2013 and July 2014. All patients then underwent physical and Laboratory examinations. The inclusion criteria were as follows: no history of myocardial infarction, heart failure, cardiac valve disease, severe renal function impairment [defined by an estimated glomerular filtration rate (eGFR) <60 ml/min/1.73 m^2^], coronary bypass surgery or angioplasty, diabetes mellitus or renal insufficiency; no treatment with urate-lowering medication (allopurinol and probenecid); no secondary or malignant hypertension. Sixty-two patients who did not meet the above criteria were excluded. Therefore, 344 hypertensive patients were involved the final statistical analysis (S1 Dataset). The study protocol was approved by the Ethics Committee of Southern Medical University, and written informed consent was obtained from all participants.

### Blood pressure measurements

Newly diagnosed hypertension was defined as systolic blood pressure (SBP) ≥ 140 mmHg and (or) diastolic blood pressure (DBP) ≥ 90 mmHg. Blood pressures were measured using a mercury sphygmomanometer. Three measurements were taken at a 10-min interval and then averaged to define the clinic SBP or DBP.

### Laboratory examinations

Serum total bilirubin, direct bilirubin, and indirect bilirubin levels were measured by the vanadate oxidation method using automatic biochemical analyzer. Hematologic test was measured using an automated hematology analyzer (Bayer Diagnostics, Newbury, and Berkshire, UK). During blood routine test [red blood cell (RBC) count, white blood cell (WBC) count, platelet count, hemoglobin, mean corpuscular volume and red cell distribution width (RDW)], fasting blood glucose, and creatinine, as well as the fasting serum lipid status including levels of total cholesterol (TC), LDL-cholesterol, high-density lipoprotein (HDL)- cholesterol, triglyceride (TG), and C-reactive protein (CRP) were recorded. Height and weight were measured, and the body mass index (BMI) was calculated as weight (kg) divided by height squared (m2). The eGFR was calculated as follows [17]: 186×SCr-1.154×age in years-0.203×1.210 (if black) ×0.742(if female) 36. Then CKD was defined as eGFR <60 ml/min/1.73 m^2^.

### Echocardiography

All echocardiographic tests were performed on each subject with a commercially available machine (Fair Medical Company Ltd, Matsudo, Japan) using a 2.5 Hz transducer. The overall single-dimensional left ventricular measurements and the two-dimensional views were obtained according to the American Society of Echocardiography standard [18, 19]. Left ventricular mass (LVM) was calculated as follows [20]: LVM = 1.04×0.8×{(VST_d_ × LVID_d_ × PWT_d_) 3-(LVID_d_) 3} +0.6, where IVS_d_ is diastolic interventricular septum, LVD_d_ is diastolic left ventricular dimension, and PWT_d_ is diastolic posterior wall thickness. The left ventricle mass index (LVMI) was computed as = LVM/BSA (Body Surface Area, BSA). LVH was defined as: LVMI ≥ 125 g/m2 for men and ≥ 110 g/m2 for women [21].

### Statistical analysis

The study patients were divided into an LVH group (n = 206) and a non-LVH group (138). Data were reported as mean ± standard deviation for quantitative variables and as percentages for qualitative variables. Whether a continuous variable was in normal distribution was assessed using the Kolmogorov—Smirnov test. Differences between groups were tested by Student t-test for continuous variables and by Chi-square test for categorical variables. Correlations between LVMI and other variables were calculated by Pearson’s or Spearman’s test as appropriate. The significant determinants for LVMI were detected with stepwise multiple linear regression, and the odds ratios (ORs) of independent variables were computed with stepwise multiple logistic regression. Collinear diagnostics within variables was applied before the regression modeling. Statistical analysis was performed on SPSS 19.0 (SPSS Inc, USA) with the significance level at P<0.05.

## Results

### General information of the subjects

Table 1 summarizes the demographic and clinical data of the two groups. The average ages of the two groups are 47.8±7.9 and 47.2±7.6 years old, respectively (P = 0.481). The sex ratios are not significantly different between groups. Total bilirubin and indirect bilirubin levels are both significantly lower in the LVH group than in the non-LVH group. Besides, the LVH group tends to be smokers and have higher SBP, DBP, glucose level, serum creatinine, uric acid, metabolic components, and RDW. Echocardiography shows that many indicators from the LVH group are significantly higher compared the non-LVH group. Other variables are all not different between the two groups (Table 1).

**Table 1:** Clinical characteristics of hypertension patients with and without LVH.

Parameters	Non-LVH group	LVH group	*P*
Age, y	47.8±7.9	47.2±7.6	0.481
Sex(male)	118(57.3%)	89(64.5%)	0.181
Smoking, yes	27(13.1%)	46(33.3%)	0.000
Body mass index, kg/m^2^	26.7±3.7	26.4±3.2	0.482
SBP, mmHg	150.6±10.0	155.4±13.3	0.000
DBP, mmHg	99.0±8.1	101.3±10.0	0.022
Triglyceride, mmol/dL L	1.8±0.5	1.9±0.5	0.515
HDL-cholesterol, mmol/dL	1.1±0.2	1.1±0.2	0.289
LDL- cholesterol, mmol/dL	3.2±0.8	3.1±0.8	0.926
Total cholesterol, mmol/dL	5.1±0.9	5.1±0.8	0.995
Glucose, mmol/dL	5.6±0.6	5.7±0.6	0.040
eGFR,mL/min/1.73	109.5±29.2	104.4±22.9	0.072
Serum creatine, mmol/dL	67.3±13.2	71.0±13.2	0.010
Uric acid, μmol/L	343.5±49.9	370.4±46.7	0.000
Hs-CRP, mg/dL	2.3±5.4	2.5±4.1	0.750
Alanine aminotransferase, U/L	32.2±27.7	29.5±19.5	0.325
Aspartate aminotransferase, U/L	25.1±10.3	25.1±12.0	0.968
Blood urea nitrogen, mmol/L	4.7±1.1	4.9±1.2	0.257
Metabolic components,n	1.6±1.0	1.8±1.1	0.145
Total bilibin, μmol/L	14.0±4.5	11.4±4.1	0.000
Direct bilirubin, μmol/L	3.2±2.4	3.1±2.6	0.999
Indirect bilirubin, μmol/L	10.8±4.5	8.2±4.2	0.000
White blood cell, ×10^12^/L	6.2±1.9	6.1±1.6	0.760
Red blood cell, ×10^12^/L	4.7±0.3	4.8±0.2	0.465
Red cell distribution width (%)	12.7±0.7	12.9±0.9	0.014
Hemoglobin,g/L	149.6±27.1	146.5±16.9	0.236
Blood platelet, 10^3^/mm^3^	242.6±54.1	243.3±56.0	0.923
IVST, mm	10.0±1.1	12.±1.2	0.000
LVEDd, mm	45.2±4.4	49.1±4.0	0.000
LVESd, mm	29.6±4.3	32.3±3.6	0.000
PWT, mm	10.1±1.7	11.3±1.3	0.000
Ejection fraction, %	65.8±5.3	63.5±7.3	0.000
Left ventricular mass, g	174.5±33.7	240.4±44.4	0.000
Left ventricular mass index, g/m2	92.3±12.7	128.1±18.5	0.000

### Univariate analyses

**Fig 1:**
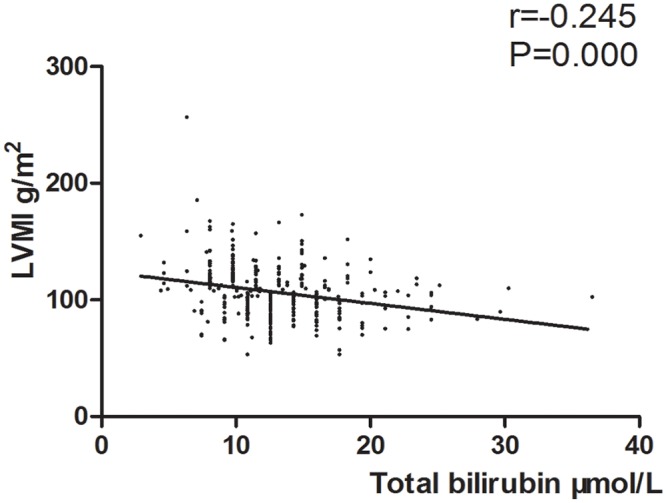
Scatter diagram of total bilirubin level between the LVMI.

**Fig 2:**
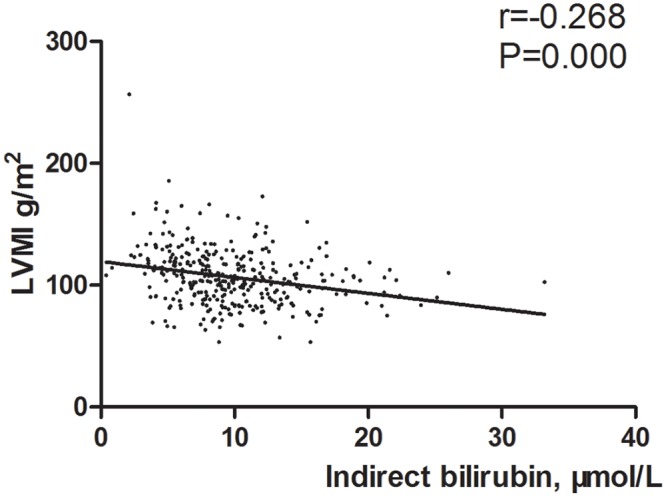
Scatter diagram of indirect bilirubin level between the LVMI.

In the whole study population, total bilirubin (Fig 1) and indirect bilirubin levels (Fig 2) are negatively associated with LVMI. Sex, smoking status, SBP, glucose, serum creatinine, uric acid, blood urea nitrogen, and RDW are positively associated with LVMI. The association between LVMI and any of other variables is not significant (Table 2).

**Table 2:** Correlation coefficients of relation between left ventricular mass index and some parameters.

Variables	Correlation coefficients	*P*
Age	-0.055	0.307
Sex	0.119*	0.027
Smoking	0.228*	0.000
SBP, mmHg	0.136	0.011
DBP, mmHg	0.089	0.101
Triglyceride, mmol/dL L	-0.005	0.922
HDL-cholesterol, mmol/dL	-0.057	0.293
LDL- cholesterol, mmol/dL	0.012	0.824
Total cholesterol, mmol/dL	0.004	0.944
Glucose, mmol/dL	0.142	0.008
eGFR, mL/min/1.73	-0.111	0.039
Serum creatine, mmol/dL	0.162	0.003
Uric acid, μmol/L	0.225	0.000
Hs-CRP, mg/dL	0.064	0.246
Alanine aminotransferase, U/L	-0.024	0.656
Aspartate aminotransferase, U/L	-0.022	0.686
Blood urea nitrogen, mmol/L	0.188	0.029
Total bilibin, μmol/L	-0.245	0.000
Direct bilirubin, μmol/L	-0.012	0.624
Indirect bilirubin, μmol/L	-0.268	0.000
White blood cell, ×10^12^/L	0.032	0.556
Red blood cell, ×10^12^/L	0.060	0.267
Red cell distribution width (%)	0.129	0.017
Hemoglobin,g/L	-0.053	0.325
Blood platelet, 10^3^/mm^3^	-0.083	0.200

*Spearman correlation coefficient

### Multiple linear regression analysis

To test whether the relation between total bilirubin level and LVMI was confounded by other factors, we conducted the multiple linear regression by considering a set of potential variables. The results suggest that total bilirubin (B = -0.017, P = 0.008) is negatively associated with LVMI, independent of age, sex, BMI, smoking status, creatinine, fasting glucose, eGFR, hsCRP, TG, HDL-cholesterol, TC, LDL-cholesterol, metabolic, alanine aminotranferease (ALT), aspartate aminotransferase (AST), blood urea nitrogen (BUN), WBC count, Plt count, RBC count, Hb, and Plt count. Table 3 shows the results of multiple linear regression performed to assess the independent predictors of LVMI.

**Table 3:** Stepwise multiple linear regression analysis for the effect of independent variables on left ventricular mass index.

Variable	*B*	S.E.	*t*	*P*
Constant	-1.135	0.443	-2.564	0.011
Smoking	0.265	0.074	3.578	0.000
Serum uric acid	0.002	0.001	3.134	0.002
Total bilirubin	-0.017	0.006	-2.695	0.008
SBP	0.007	0.003	2.557	0.011

### Multiple logistic regression analysis

In a stepwise multiple logistic regression model, LVH was defined as the dependent variable and other factors with LVH as covariates. The results show that total bilirubin [OR = 0.91, 95%CI (confidence interval): 0.85–0.97] is an independent protective factor of LVH. Other factors including smoking status (OR = 3.14, 95% CI: 1.66–7.00), serum acid (OR = 1.01, 95%CI: 1.00–1.02), and SBP (OR = 1.03, 95%CI: 1.01–1.06) (Table 4).

**Table 4:** Multivariable logistic regression analysis of prediction of LVH.

Variable	*B*	*S*.*E*.	Waldχ^2^	Odds ratio	95% CI	*P*
Constant	-8.009	2.333	11.784	-	-	0.001
Smoking	1.227	0.367	11.194	3.14	1.66–7.00	0.001
Serum uric acid	0.010	0.003	9.178	1.01	1.00–1.02	0.002
Total bilirubin	-0.090	0.035	6.625	0.91	0.85–0.97	0.010
SBP	0.032	0.013	6.143	1.03	1.01–1.06	0.013

## Discussion

The main findings of the present study are that total serum bilirubin level is negatively associated with LVMI and serves as a protective factor of LVH in hypertensive patients without receiving any drug treatment, regardless of potential confounding factors. The same protective effect of bilirubin on LVH was observed in a hypertensive rat model [22]. In a recent report involving 114 consecutive hypertensive patients, bilirubin level is positively related with LVMI and LVH [23], which is totally different from our results. Compared with their study, our findings have several strengths that need to be addressed. On one hand, the present study involves a sample size three times larger than theirs (344 vs. 114). Moreover, their logistic regression results show quite a wide CI (1.035–12.846). The small sample size is likely to induce larger sampling error, which reduces the precision of the results. On the other hand, hypertensive patients with diabetes mellitus were excluded from our study. About 10.5% of the study population had diabetes mellitus, and most of them tended to be obesity (BMI: 31.7±5.3), which may have modest effect on our results. Thus, we extended the previous findings using a larger sample size and established that reduction in bilirubin levels may precede the development of LVH in untreated hypertensive patients.

As a major product of heme metabolism in the vessels, serum bilirubin could be poisonous for infants under excessive condition [24], but a potential antioxidant material can provide profitable protection against CVDs [25]. Actually, many epidemiologic studies demonstrate that higher serum bilirubin level is a protective factor of CVDs. As reported, serum bilirubin level is independently and negatively associated with the occurrence of carotid atherosclerosis in both males and females [26]. The causal risk reduction for type 2 diabetes (T2DM) was estimated to be 42%, which was comparable to the observational estimate [27]. This result provides strong evidence that the elevated bilirubin level is significantly associated with the reduced risk of T2DM and supports its role as a protective determinant. A prospective large-scale and community-based cohort with 8593 Korean patients shows that lower bilirubin level is an risk factor of coronary artery disease [adjusted hazard ratio:1.89 [28]. Our results support the current opinion. The exact mechanism about the association between serum bilirubin level and LVH remains unclear, but there are several possible reasons. First, bilirubin is an antioxidant, which has been confirmed in vitro and in vivo [29, 30]. Human trials also suggest that bilirubin level is positively related with the total antioxidant capacity [31]. It is generally suggested that oxidative reactions are involved in the pathophysiology of CVDs [32]. Bilirubin can suppress the oxidative modification of LDL-lipoprotein, an important part of the development of atherosclerosis. Second, serum bilirubin may be associated with LVH through the close relation with regular risk factors. It is suggested that high bilirubin levels are negatively associated with the presence of obesity, smoking status, diabetes mellitus, hypertension and metabolic syndrome. Finally, the anti-inflammatory function of bilirubin can partly explain why bilirubin level is lower in the LVH group. Animal experiments prove that the intake of bilirubin can affect the expressions of cell adhesion molecules [33]. Besides, an epidemiological study also reveals the inverse relation between bilirubin and hs-CRP [34]. Nevertheless, further studies are needed.

The present study has several limitations. First and most importantly, the nature of cross-sectional design does not allow us to confirm the cause-and-effect relation. Actually, the serum bilirubin level is very valuable as a reference in clinic, no matter the lower serum bilirubin is a cause or effect of LVH. After all, the serum bilirubin level could be thought as a relative index to LVH. Second, some inflammation markers (interleukin-6 and -10) were not reported. Bilirubin without other clinical index may not provide enough information for clinicians. Third, it should be noted that our findings are based on the selected Asian population with moderate primary hypertension. Patients with CKD or cardiovascular diseases were excluded from this study. Therefore, the extrapolation to other populations will be limited. Nevertheless, this point makes our results independent from some confounders. Finally, although our results prove an inverse association different from previous research, the specific mechanism cannot be illustrated clearly. Therefore, further studies regarding the role of serum bilirubin on the development of LVH are needed.

In conclusion, total serum bilirubin level is inversely associated with the development of LVH in the untreated hypertensive patients after adjustment of some potential confounders. Thus, as a routine quick laboratory examination index, serum bilirubin level may be treated as a novel marker for evaluation of target organ damage risk (especially LVH) in hypertensive patients. Subsequent studies should focus on the exact biological mechanism.

## Supporting Information

S1 Dataset Original data file. (XLS)
